# Clinical and genetic characteristics of patients diagnosed with atypical hemolytic uremic syndrome (aHUS): epidemiological data from the Belgian cohort of the Global aHUS Registry

**DOI:** 10.1007/s40620-025-02366-7

**Published:** 2025-10-17

**Authors:** Annick Massart, Laurent Weekers, Kathleen J. Claes, Tess Van Meerhaeghe, Evelien Snauwaert, Djalila Mekahli, Eric Goffin, Laure Collard, Nathalie Godefroid, Brigitte Adams, Stefan Van Cauwelaert, Koenraad Van Hoeck, Sebastien Block, Imad Al-Dakkak, Karin Dahan, Patrick Stordeur, Johan Vande Walle

**Affiliations:** 1 Department of Nephrology, Antwerp University Hospital, Antwerp, Belgium; 2 Department of Nephrology, University of Liège, CHU Liège, Liège, Belgium; 3 Department of Nephrology and Renal Transplantation, Leuven University Hospital, Louvain, Belgium; 4 KU Leuven, Department of Microbiology and Immunology, Laboratory of Nephrology, Louvain, Belgium; 5 Department of Nephrology, Hôpital Universitaire de Bruxelles, Erasme, Brussels, Belgium; 6 Department of Pediatric Nephrology, Ghent University Hospital, Ghent, Belgium; 7 Department of Pediatric Nephrology and Organ Transplantation, Leuven University Hospitals, Louvain, Belgium; 8 PKD Research Group, Laboratory of Ion Channel Research, Department of Cellular and Molecular Medicine, KU Leuven, Louvain, Belgium; 9 Department of Nephrology, Cliniques universitaires Saint-Luc, Université Catholique de Louvain, Brussels, Belgium; 10 Pediatric Department, CHC Montlégia, Liège, Belgium; 11 Pediatric Department, Cliniques universitaires Saint-Luc, Université Catholique de Louvain, Brussels, Belgium; 12 Queen Fabiola Children’s University Hospital, Brussels, Belgium; 13 Department of Nephrology and Transplantation, Universitair Ziekenhuis Brussels, Brussels, Belgium; 14 Department of Pediatrics, Antwerp University Hospital, Antwerp, Belgium; 15 Alexion Pharma Belgium, Brussels, Belgium; 16 Global Epidemiology, Alexion Pharmaceuticals, Inc, Boston, MA USA; 17 Institut de Pathologie et de Génétique, Centre de Génétique, Gosselies, Belgium; 18 Laboratoire d’Immunologie (LHUB), Belgian National Reference Center for the Complement System, Hôpital Universitaire de Bruxelles, Brussels, Belgium

**Keywords:** aHUS Registry, Epidemiology, Prevalence, Gender

## Abstract

**Background:**

Atypical hemolytic uremic syndrome (aHUS) usually results from an overactivation of the alternative complement pathway. As large clinical trials are scarce, patient registries can partially fill the knowledge gap on patient characteristics, management, and outcomes. We here describe the baseline clinical and genetic characteristics as well as the management of all Belgian patients enrolled in the Global aHUS Registry at data cut-off.

**Methods:**

This observational study prospectively and retrospectively collected data (data cut-off: December 26, 2022) from patients of all ages with a clinical diagnosis of aHUS, irrespective of treatment.

**Results:**

A total of 121 Belgian patients were registered in the Global aHUS Registry, resulting in a prevalence of 10.4 aHUS patients per million inhabitants, with a higher proportion of females affected (57.9% vs 42.1% of males). Among the 109 patients tested for at least one variant and/or anti-complement factor H (CFH) antibodies, 36 were positive for a pathogenic complement gene variant associated with aHUS (*n* = 29) and/or seropositive for anti-CFH antibodies (*n* = 14). The most common variants affected *CFH*, *C3* and *CD46*. The higher proportion of complement gene variants in treated women versus men was not related to a specific gene.

**Conclusions:**

This study strengthens the real-world evidence on aHUS and adds to previously published Global aHUS Registry data. In addition, it provides insights into the differential epidemiology of the disease in Belgium and demonstrates the increased susceptibility of women to aHUS across the whole spectrum of recognized complement gene variants.

**Graphical abstract:**

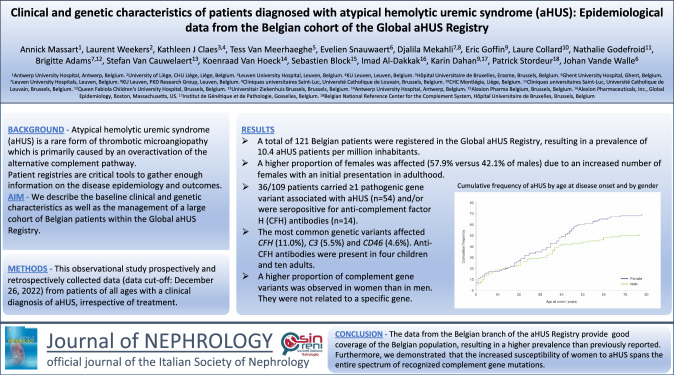

**Supplementary Information:**

The online version contains supplementary material available at 10.1007/s40620-025-02366-7.

## Introduction

Atypical hemolytic uremic syndrome (aHUS) is characterized by a triad of thrombocytopenia, mechanical hemolytic anemia, and predominantly acute kidney injury, although the heart, brain, lungs, and gastrointestinal tract may be involved [1]. Due to the large clinical overlap between the different thrombotic microangiopathies (TMAs), the diagnosis of aHUS relies on the stepwise elimination of other forms of TMAs including thrombotic thrombocytopenic purpura (TTP), cobalamin C defect, Shiga toxin-producing *Escherichia coli*-HUS, pneumococci-mediated HUS, and obvious secondary HUS [2].

Most cases of aHUS (40–70%) result from a hereditary or acquired unhampered activation of the alternative complement pathway [3, 4]. This can arise from loss-of-function genetic variants affecting complement regulators such as complement factor (CF) H, CFI, membrane cofactor protein (CD46), and thrombomodulin (THBD); from activating variants in critical complement components such as complement component 3 (C3) and CFB; or from autoantibodies targeting CFH [5, 6]. Approximately 30% of aHUS arise from unknown mechanisms [7]. aHUS often occurs in the setting of, or is triggered by, certain conditions, including malignant hypertension, autoimmune diseases, cancers, use of various medications, transplantation, or common infections [6, 7].

Prior to the availability of complement inhibitors, patients diagnosed with aHUS were treated with plasma exchange (PE), resulting in poor outcomes. Approximately 56% of adults and 29% of children progressed to kidney failure (KF) or died within one year following the first clinical manifestation [3, 7]. Moreover, the disease relapsed after kidney transplantation in approximately 60% of patients, causing graft failure in 92% [8]. The use of complement inhibitors, namely the anti-C5 monoclonal antibody eculizumab, has revolutionized the management and improved the prognosis of aHUS patients [9, 10]. As with other rare diseases, large and sufficiently powered aHUS-related clinical trials are scarce. Therefore, international patient registries are needed to achieve a sufficient sample size to inform about the natural history of aHUS, management, and outcomes.

In 2012, the Global aHUS Registry was initiated at the request of the United States (US) Food and Drug Administration and the European Medicines Agency, aiming at capturing the natural history of aHUS patients, whether treated or not, as well as providing pharmacovigilance data following the commercialization of eculizumab, and later, ravulizumab. The Registry enrolled more than 2100 patients from 15 countries across four continents and is the largest database of aHUS patients in the world to date. Several studies have been published based on these data and provided important epidemiological information [11, 12].

In this study, we describe the baseline clinical and genetic characteristics and management of all Belgian patients in the Global aHUS Registry at data cut-off. These nationwide data may provide significant contribution to the knowledge of aHUS natural history and epidemiology.

## Methods

### Study design and population

Details of the methodology and objectives of the Global aHUS Registry (NCT01522183) managed by Alexion Pharmaceuticals, Inc. (New Haven, CT, US) were previously reported [11]. Briefly, the Global aHUS Registry is an international observational study initiated in 2012 that prospectively and retrospectively collects data from patients of all ages with a clinical diagnosis of aHUS, irrespective of treatment. The identification of an underlying complement defect was not a prerequisite for enrollment. Exclusion criteria were HUS only due to Shiga toxin and ADAMTS13 activity ≤ 5%, if performed.

The Global aHUS Registry included nine Belgian sites: eight academic centers and one general hospital (Online Resource 1).

### Data collection and analysis

The Registry was designed to collect and evaluate safety and effectiveness data on eculizumab and ravulizumab use in aHUS patients, and to assess long-term manifestations of aHUS complications, regardless of treatment approach. The management of patients was not influenced by their inclusion in the registry and was left to the sole discretion of the treating nephrologist, within the framework of local regulatory constraints (Online Resource 2). The following patient data were collected at enrollment and every six months thereafter: demographic characteristics, disease history and potential triggering conditions from a preselected list (including: autoimmune disease [systemic lupus erythematosus, scleroderma, or anti-phospholipid syndrome], pregnancy, malignancy and malignant hypertension, and prior kidney transplantation), symptomatology, investigator-reported TMA complications, relevant laboratory results (including genetic and serological testing when performed), aHUS targeted treatments and concomitant medications, clinical and patient-reported outcomes, and safety evaluations. Data were collected from patient medical records and entered in a web-based secure electronic data collection system. Data cut-off date for the present study was December 26, 2022.

TMA occurrence and relapse rates were evaluated in patients diagnosed with aHUS before 2011 who were followed up for at least six months. Vital organ manifestations were collected for all patients preceding study entry.

If any, variants identified in aHUS-associated genes were recorded (*C3*, *CFH*, *CFI*, *CFB*, *CD46*, *DGKE*, and *THBD*) as well as the presence of anti-CFH antibodies. All variants were individually reviewed by an internationally recognized expert geneticist, specialized in aHUS and with longstanding expertise in the field (Pr. V. Frémeaux-Bacchi). A variant was defined as rare if its minor allele frequency was below 0.1% in the European population using the GnomAD data base (gnomAD - Non-Finnish European [NFE], https://gnomad.broadinstitute.org). Rare variant was defined as pathogenic if the genetic change affected the protein function (well-established *in vitro* functional studies supportive of a damaging effect on the gene product), and/or if the genetic change was found in a disease-related functional domain, and/or affected the protein expression (well demonstrated lack of *in vitro* synthesis, large deletion or quantitative deficiency in the patient’s plasma). The other variants were classified as variants of uncertain significance (VUS).

The results of genetic and serology testing were categorized as: (i) presence of any rare pathogenic aHUS-associated gene variants or seropositivity for anti-CFH antibodies; (ii) no identified rare pathogenic variant and seronegativity for anti-CFH antibodies among patients tested for ≥ 5 genes; (iii) inconclusive (patients without an identified variant but for whom not all relevant genes were tested, or patients with an ambiguous genetic report). Complement protein analysis included assessment of C3, C4, FH, FI, and CD46. Sequencing and dosing methods were not recorded in the database.

### Statistical analysis

Patient data were analyzed using descriptive statistics. Categorical variables were computed as numbers and proportions (in percentages), while continuous variables were computed as mean, median and interquartile range (IQR) values. Family history of aHUS was defined as presence of ≥1 family member with aHUS for a given patient. Data were stratified according to age at initial presentation (childhood [<18 years], adulthood [≥18 years], and overall) and gender. Cumulative age at aHUS onset was evaluated using Kaplan-Meier estimates. Cox-proportional hazards model was used to evaluate important risk factors (age at initial aHUS presentation, gender, family history of aHUS, time from initial aHUS presentation to diagnosis, and presence of triggering factors) associated with KF (hazard ratios [HR] with 95% confidence interval [CI]). All statistical analyses were done using SAS (version 9.2, SAS Institute, Cary, NC, US).

## Results

### Population of Belgian patients diagnosed with aHUS in the Registry

On December 26, 2022, 121 Belgian patients with aHUS were enrolled in the Registry, leading to a prevalence of 10.4 aHUS patients per million inhabitants.

**Fig. 1:**
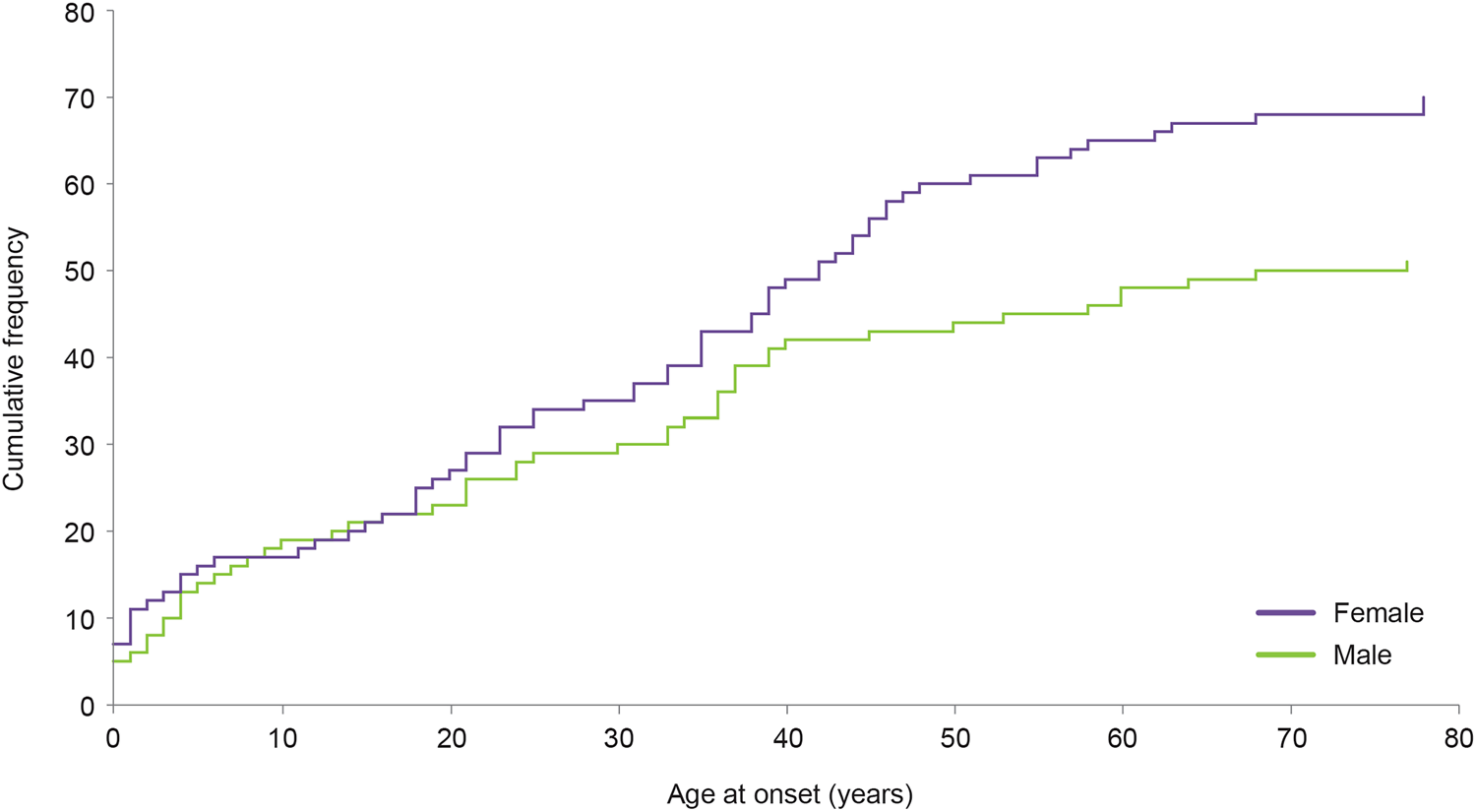
Cumulative frequency of aHUS by age at disease onset and by gender. *aHUS* atypical hemolytic uremic syndrome

Among the 121 patients enrolled, 77 were adults and 44 were children; 70 (57.9%) were female (Table 1). The higher proportion of females resulted from an increased number of females with an initial presentation in adulthood (62.3% of adults vs 50.0% in children). This is also reflected in the cumulative frequency of aHUS by gender (Fig. 1).

**Table 1:** Baseline patient demographics, clinical characteristics, and treatment

Characteristics	Total (*N* = 121)	Initial presentation in adulthood (*N* = 77)	Initial presentation in childhood (*N* = 44)
**Baseline**			
Age (years), median (IQR)			
At 1st aHUS manifestation	25.1 (6.5–40.6)	38.6 (28.4–47.4)	3.6 (0.7–7.7)
Sex, *n* (%)			
Female	70 (57.9)	48 (62.3)	22 (50.0)
Male	51 (42.1)	29 (37.7)	22 (50.0)
Family history of aHUS, *n* (%)			
Yes	17 (14.0)	10 (13.0)	7 (15.9)
No	78 (64.5)	45 (58.4)	33 (75.0)
Unknown	26 (21.5)	22 (28.6)	4 (9.1)
TMA relapse between disease diagnosis and enrollment/eculizumab treatment initiation, *n* (%)	59 (48.8)	31 (40.3)	28 (63.6)
Disease duration^a^ (years), median (IQR)	0.9 (0.1–6.5)	0.2 (0.0–3.8)	2.9 (0.6–8.9)
Patients with 1st aHUS manifestation before 2011, *n* (%)	32 (26.4)	18 (23.4)	14 (31.8)
Dialysis prior to baseline^b^, *n* (%)	67 (55.4)	47 (61.0)	20 (45.5)
Chronic dialysis (duration > 3 m) at baseline^b^, *n* (%)	47 (38.8)	35 (45.5)	12 (27.3)
Plasma exchange/infusion prior to baseline^b^, *n* (%)	86 (71.1)	60 (77.9)	26 (59.1)
Duration of plasma exchange/infusion (m), median (IQR)	0.4 (0.2–1.0)	0.5 (0.2–1.2)	0.1 (0.1–0.4)
Kidney transplantation prior to baseline^b^, *n* (%)	23 (19.0)	17 (22.1)	6 (13.6)
Number of transplants, *n* (%)			
1	17 (14.0)	14 (18.2)	3 (6.8)
≥ 2	6 (5.0)	3 (3.9)	3 (6.8)
Time from aHUS onset to treatment (years), median (IQR)	0.1 (0.0–2.8)	0.1 (0.0–1.6)	0.1 (0.0–5.2)
**Treatment**			
Eculizumab treatment at any time, n (%)	63 (52.1)	41 (53.2)	22 (50.0)
Duration of eculizumab treatment (m), median (IQR)	6.0 (2.1–15.2)	6.0 (3.7–12.7)	5.5 (0.7–22.7)
Plasma exchange/infusion at any time, *n* (%)	93 (76.9)	65 (84.4)	28 (63.6)
Kidney transplantation at any time, *n* (%)	33 (27.3)	23 (29.9)	10 (22.7)

*N* number of patients; *IQR* interquartile range; *aHUS* atypical hemolytic uremic syndrome; n/%, number/percentage of patients in a given group; *TMA* thrombotic microangiopathy; m, months

^a^from initial presentation to enrollment

^b^baseline was defined as enrollment for untreated patients and treatment initiation for treated patients

The median age at disease onset was 25.1 (IQR 6.5–40.6) years; 38.6 (28.4–47.4) years for adults and 3.6 (0.7–7.7) years for children. A minority of patients (14.0%) had a family history of aHUS (Table 1). Between aHUS diagnosis and enrollment in the Registry, 59 patients (31 [40.3%] adults and 28 [63.6%] children) experienced a TMA relapse as defined by the investigator (Table 1). As per protocol, no patient had an ADAMTS13 activity below 5%, nor between 5% and 10%.

### Patient treatment and outcomes

The management of aHUS was PE and/or plasma infusion (PI) (PE/PI) during the course of the disease in 93 patients (65 [84.4%] adults and 28 [63.6%] children), and 63 patients were treated with eculizumab (41 [53.2%] adults and 22 [50.0%] children) (Table 1). The median duration of PE/PI was 0.3 (IQR 0.1–0.9) months (0.5 [0.2–0.9] months in adults and 0.2 [0.1–0.4] months in children) in the eculizumab-treated group and 0.4 (0.2–1.5) months (0.6 [0.4–2.0] and 0.2 [0.0–0.4] months, respectively) in the group that was never treated with eculizumab (never-treated group). The median duration of eculizumab treatment was 6.0 (2.1–15.2) months (6.0 [3.7–12.7] months in adults and 5.5 [0.7–22.7] months in children) (Table 1).

A total of 49 (40.5%) patients required kidney replacement therapy for KF (37 [48.0%] adults and 12 [27.3%] children), consisting of chronic dialysis (>3 months) in 47 (38.8%) patients (35 [45.5%] adults and 12 [27.3%] children) and/or kidney transplantation in 33 (27.3%) patients (23 [29.9%] adults and 10 [22.7%] children) (Table 1).

### Genetic variants, complement system evaluation, and associated conditions

In total, 109 (90.1%) patients - 70 adults and 39 children - were tested for at least one complement gene variant and/or presence of serum anti-CFH antibodies, and 80 (66.1%) were tested for at least five variants (Online Resource 3). In the entire cohort, 36 (29.8%) patients were positive for at least one pathogenic variant (*n* = 29) and/or seropositive for anti-CFH antibodies (*n* = 14). The most frequent pathogenic variants identified among tested patients were in *CFH* (12 [11.0%] patients), *C3* (6 [5.5%]), and *CD46* (5 [4.6%]). Anti-CFH antibodies were present in ten adults and four children (Online Resources 3 and 4). Other variations were observed but could not be retained, namely: VUS [4 in *CFH*, 2 in *CD46* and 1 in *C3*]; polymorphisms falsely reported as pathogenic; and ambiguous results [together: 9 in *CFH*, 12 in *CD46*, 5 in *C3* and 4 in *CFI*].

Eculizumab-treated and never-treated groups differed in several aspects. A higher proportion of treated patients were tested for at least one complement gene variant or anti-CFH antibodies (60 [95.2%] vs 49 [84.5%] in the never-treated group). Accordingly, percentages of patients positive for complement gene variants and anti-CFH antibodies were higher in the eculizumab-treated group (34.5% and 15.9% vs 12.1% and 6.9% in the never-treated group). *CFH*, *C3* and *CFI* were the most frequent variant-carrying genes in eculizumab-treated adults (17.0%, 9.8%, and 4.8%, respectively) while *CFH*, *CD46* and *DGKE* variants were the most frequent among treated children (18.1%, 9.1%, and 4.5%). *CD46* and *C3* variants were the most frequent variant-carrying genes (3.4% for each) in never-treated adults (Online Resources 3 and 4).

Looking further at the eculizumab-treated group, with the highest prevalence of complement gene variants and anti-CFH antibodies, we observed that females were more often tested than males for at least one gene variant or anti-CFH antibodies (29 [70.7%] vs 10 [24.4%] in adults, 12 [54.5%] vs 9 [40.9%] in children). Accordingly, variants were more often detected in females (12 [29.2%] vs 3 [7.3%] in adults, 4 [18.1%] vs 3 [13.6%] in children). Among tested patients, the proportion of females who tested positive for at least one variant or anti-CFH antibodies was markedly higher in adults (54.5% (*n* = 12/22) of females vs 30% (*n* = 3/10) of males) but not in children (33.3% for both groups). The higher proportion of complement gene variants in treated women compared to men was not related to a specific gene, and was observed for *CFH* (5 [12.2%] vs 2 [4.2%]), *C3* (3 [7.3%] vs 1 [2.4%]), *CFI* (3 [7.3%] vs 0), and anti-CFH antibodies (7 [17.1%] vs 1 [2.4%]).

Serum concentrations of C3 and C4 were assessed in most patients (111 [91.7%] and 101 [83.5%]), while serum levels of FH and FI, and the assessment of expression of CD46 on white blood cells was less commonly examined (in 76 [62.8%], 67 [55.4%], and 41 [33.9%] of patients); these results were normal in most patients (C3: 57.9%, C4: 62.8%, FH: 47.1%, FI: 47.9%, and CD46: 28.1%).

Precipitating events and/or associated conditions issued from a preselected list were only reported for a minority of patients (21 [27.3%] adults and 3 [6.8%] children) (Online Resource 5). Malignant hypertension was the only associated condition reported among children, while prior transplant, malignancy, pregnancy, autoimmune disease, and malignant hypertension were declared in adults (Online Resource 5). Precipitating factors were relatively more frequent in eculizumab-treated than never-treated adults (13 [31.7%] vs 8 [22.2%]). In the eculizumab-treated group, most precipitating factors were noticed among women (12 vs 1 case in a male) (Online Resource 5).

### Kidney failure risk factors and outcomes

**Fig. 2:**
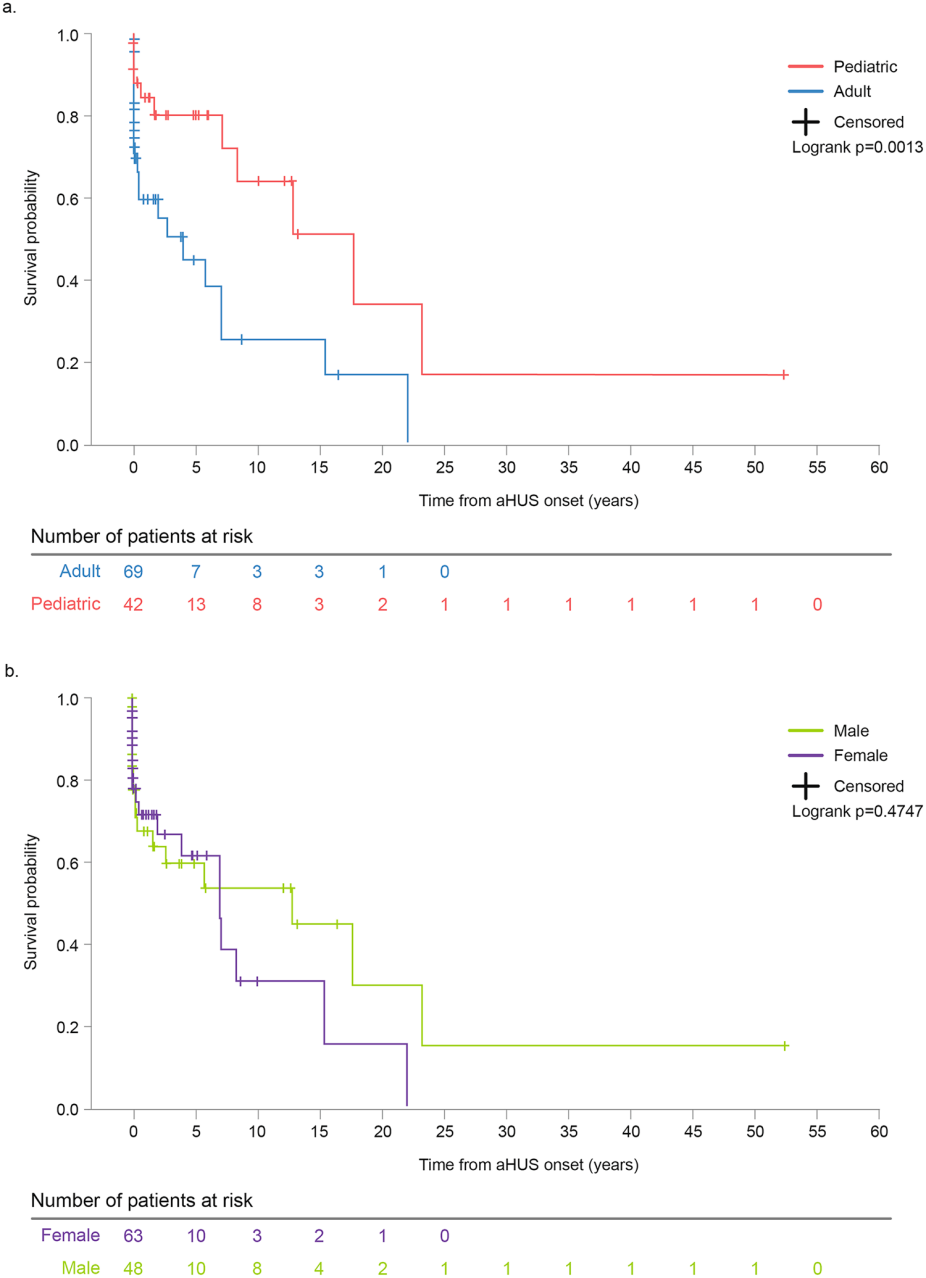
Cumulative Kaplan–Meier estimates for KF-free survival in adults and children with aHUS (**a**) and by gender (**b**). *KF* kidney failure; *aHUS* atypical hemolytic uremic syndrome

Kaplan-Meier estimates for KF-free survival from disease onset showed that a disease presentation in adulthood increased the risk of developing KF at 5 years (adjusted HR: 0.2 [95% CI: 0.1–0.6]) (Online Resource 6). Adults demonstrated a KF-free survival probability of 0.5 at 5 years vs 0.8 for children (*p* = 0.001). The other factors assessed, i.e., age, gender, race, family history of aHUS, and time from disease onset to diagnosis showed no statistically significant differences. Cumulative Kaplan-Meier estimates of patient age at disease onset and gender are shown in Fig. 2a and b, respectively.

## Discussion

In this study, we present Belgian data from the Global aHUS Registry comprising a population of 121 patients. Our findings largely corroborate previous global and non-Belgian observations on the clinical features of aHUS and also provide new epidemiological insights into the disease.

First, we established the prevalence of aHUS in Belgium as at least 10.4 aHUS patients per million inhabitants, which is the highest among all countries included in the Global aHUS Registry [13]. This could be due to the increased awareness about aHUS and access to diagnostic testing in Belgium, the center coverage in the country (i.e., more registry centers per person compared to other countries), or the interest of Belgian nephrologists. Given the voluntary registration of patients, this represents the minimal prevalence of the disease in Belgium. This also suggests that prevalences in other countries were probably underestimated [14, 15]. With a median age at presentation of 25 years and considering that only a minority of patients (<15%) had recognized familial history of aHUS, as also confirmed in other studies [3, 11, 12, 14–17], aHUS may not only concern pediatricians, nephrologists, or geneticists, but also general practitioners and various specialists.

Second, while the higher proportion of aHUS among post-puberty females was already described elsewhere [3, 12, 14, 15, 17], our study shows that the increased susceptibility of Belgian women to aHUS extends beyond a specific variant or trigger. The higher proportion of women with aHUS reflects an increase across the full spectrum of traditional variants and recognized triggers and does not seem limited to pregnancy complications. The relative contributions of endogenous estrogen production, hormonal contraception and/or pregnancies in these observations [16, 18, 19] would deserve further investigations, although these data were not available for the present study.

Third, a high completeness of genetic and biochemical testing is achieved in Belgium, with 90.1% of patients tested for at least one gene and/or anti-CFH antibodies and 66.1% for at least five genes, compared to 50% in the Global Registry [12] and 32% in Germany [17]. Among the patients tested for at least one variant and/or anti-CFH antibodies, 33.0% (*n* = 36/109) were positive for a pathogenic complement gene variant associated with aHUS (*n* = 29) and/or seropositive for anti-CFH antibodies (*n* = 14). The most common variants affected *CFH* (11.0%), followed by *C3* (5.5%) and *CD46* (4.6%), in line with previous studies [3, 4, 12, 20], although the frequency of *CFH* variants was lower in our cohort. This may be attributable to our stringent variant selection process, established by an international aHUS genetics expert, excluding polymorphisms, VUS, and any ambiguously reported variant. Additionally, due to our inclusion criteria, the registry included patients with very heterogeneous backgrounds, ranging from confirmed complement-mediated TMAs to various secondary TMAs not driven by bacterial infection or ADAMTS13 deficiency. When focusing on patients treated with eculizumab –reimbursement for which in Belgium requires rigorous exclusion of all secondary TMAs[21] – the prevalence of *CFH* variants increases toward expected levels (11 *CFH* variants among 60 patients tested for at least one gene, i.e. 18.3%).

In our study as in others, complement biochemical testing appears less sensitive than genetics [1]. aHUS can therefore not be ruled out on this basis. The discrepancies between genetic and biochemical testing account for at least two phenomena: first, a great deal of serological complement testing normalized between flares [22]; second, many complement gene variants lead to dysfunctional proteins without quantitative deficiencies [22–24]. Dysfunctional proteins are not amenable to simple biochemical dosages. Despite its lower sensitivity, biochemical complement testing may still help demonstrate an anomaly in the alternative complement pathway before the genetic tests become available. In our study, we observed a lower rate of testing for CD46 expression on blood cells. This may result from logistical problems as the analysis must be performed timely (within six hours) on a fresh blood sample in a centralized laboratory.

The severity of the disease, as indicated by the high proportions of patients requiring dialysis (55.4%) and plasma therapy (71.1%) prior to baseline, is similar to previously published reports [12, 14, 15, 17], highlighting the need for a prompt and appropriate therapy with complement inhibitors. Children were at a lower risk of developing KF, as previously reported [3, 12, 14]. This may be due to physiological aspects, the higher incidence of CD46-mediated aHUS, and/or different clinical practices as children may be referred sooner, receive a prompter diagnosis, and undergo more aggressive treatment than adults [12] .

Our study has several limitations inherent to registry-based research. First, selection bias may have occurred due to the non-random inclusion of patients. Although our cohort size is relatively large compared to similar studies, it does not represent the entire population with aHUS. Inclusion depended on both recognition by clinicians—which is challenging due to the heterogeneity of aHUS presentations—and patient willingness to participate. Especially, the lack of a standardized diagnostic test and the ongoing debates around aHUS definitions [25, 26] meant that inclusion relied heavily on the clinical judgment of investigators, which likely varied across centers. This may have influenced the proportions of primary complement-mediated aHUS versus secondary thrombotic microangiopathies. To minimize this, we analyzed separately patients who received eculizumab and those who did not, as treatment was restricted by Belgian regulations to patients with clear complement involvement. Secondly, the observationnal nature of the regsitry together with the lack of a control group limited our ability to draw conclusions about treatment efficacy.

In conclusion, this study further strengthens the real-world evidence on aHUS. The number of enrolled Belgian patients is higher than in many other countries [14, 15] and adds epidemiological value to the previously published Global aHUS Registry data. With 121 patients enrolled for an overall population of 11.69 million, the Belgian branch of the Global aHUS Registry contributes to one of the highest aHUS prevalence rates detected to date [13]. Furthermore, our analysis by gender emphasizes the differential epidemiology of aHUS in males and females and demonstrates the increased susceptibility of females to aHUS across the whole spectrum of recognized complement gene variants. Further research is warranted to understand the gender-specific risk factors for aHUS in women.

## Supplementary Information

Below is the link to the electronic supplementary material.

Supplementary file1 (DOCX 174 KB)

## Data Availability

The datasets generated during and/or analysed during the current study are available from the corresponding author on reasonable request.
