# Supplementary material for: Clinical and genetic characteristics of patients diagnosed with atypical hemolytic uremic syndrome (aHUS): epidemiological data from the Belgian cohort of the Global aHUS Registry
Source: J Nephrol. 2025 Oct 17;38(9):2841–50. doi: 10.1007/s40620-025-02366-7 (PMC12711931; doi:10.1007/s40620-025-02366-7)
Supplement: Supplementary file 1 — Supplementary file1 (DOCX 174 KB) [file 40620_2025_2366_MOESM1_ESM.docx]

**Clinical and genetic characteristics of patients diagnosed with atypical hemolytic uremic syndrome (aHUS): Epidemiological data from the Belgian cohort of the Global aHUS Registry**

Annick Massart, Laurent Weekers, Kathleen J Claes, Tess Van Meerhaeghe, Evelien Snauwaert, Djalila Mekahli*,* Eric Goffin, Laure Collard, Nathalie Godefroid, Brigitte Adams, Stefan Van Cauwelaert*,* Koenraad Van Hoeck, Sebastien Block, Imad Al-Dakkak, Karin Dahan, Patrick Stordeur, Johan Vande Walle

# Supplementary Material

**Online Resource 1**

**Supplementary Methods - Study design**

Belgian centers included in the study:

1. Liège University Hospital (Centre Hospitalier Universitaire de Liège [CHU Liège]) – adult center
2. Leuven University Hospital (Universitair Ziekenhuis Leuven [UZ Leuven]) – adult and pediatric centers
3. Hospital Erasme (Hôpital Erasme - Cliniques Universitaires de Bruxelles) – adult center
4. Antwerp University Hospital (Universitair Ziekenhuis Antwerpen [UZA]) – adult and pediatric centers
5. Cliniques Universitaires Saint-Luc – adult and pediatric centers
6. Brussels University Hospital (Universitair Ziekenhuis Brussel [UZ Brussel]) – adult center
7. CHC Montlegia, Liège – pediatric center
8. Queen Fabiola Children's University Hospital (Hôpital Universitaire des Enfants Reine Fabiola) – pediatric center.
9. Ghent University Hospital (Universitair Ziekenhuis Gent [UZ Gent]) – pediatric center

**Online Resource 2**

**Treatment allocation and termination**

**Allocation - Given the observational design of this study,** therapeutic decisions were left to the discretion of the treating nephrologists, within the framework of existing regulatory constraints. Broadly, four main therapeutic trajectories could be identified among the patients included in the registry.

*Note: Eculizumab has been reimbursable in Belgium since 2013.*

Termination - In Belgium, eculizumab reimbursement is granted for a given period (12 months in between 2013 and 2017 and 6 months since 2017). Extensions of one year are possible, but are each time subject to a case-by-case assessment by a college of experts who take into account the genotyping and the past medical history of the patient. Treatment termination before 6 months was allowed but left to discretion to the discretion of each center.

**Online Resource 3**

Complement gene variants and anti-CFH antibody seropositivity at initial presentation by age (eculizumab-treated group)

| **Eculizumab-treated** | | | | | | | | | | | | | | **Treated and never-treated** |  |
| --- | --- | --- | --- | --- | --- | --- | --- | --- | --- | --- | --- | --- | --- | --- | --- |
|  | **Initial presentation in adulthood** | | | | | **Initial presentation in childhood** | | | | |  | **Adults and children** |  | **Adults and children** | |
| **Variable, n (%)** | **Male** | | **Female** | | **Total** | **Male** | | **Female** | | **Total** |  |  |  |  |  |
|  | **N=11** | **% of all adults** | **N=30** | **% of all adults** | **N=41** | **N=10** | **% of all children** | **N=12** | **% of all children** | **N=22** |  | **N=63** |  | **N=121** | |
| Tested for ≥5 variants | 9 (81.8) | 22.0 | 23 (76.7) | 56.1 | 32 (78.0) | 5 (50.0) | 22.7 | 10 (83.3) | 45.5 | 15 (68.2) |  | 47 (74.6) |  | 80 (66.1) | |
| Tested for any variant (≥1) or anti-CFH Ab | 10 (90.9) | 24.4 | 29 (96.7) | 70.7 | 39 (95.1) | 8 (80.0) | 36.4 | 12 (100.0) | 54.5 | 20 (90.9) |  | 59 (93.7) |  | 108 (89.3) | |
| Tested positive for ≥1 variant | 4 (36.4) | 9.8 | 18 (60.0) | 43.9 | 22 (53.7) | 4 (40.0) | 18.2 | 6 (50.0) | 27.3 | 10 (45.5) |  | 32 (50.8) |  | 54 (44.6) | |
| Tested positive for ≥1 variant or anti-CFH Ab | 4 (36.4) | 9.8 | 19 (63.3) | 46.3 | 23 (56.1) | 5 (50.0) | 22.7 | 6 (50.0) | 27.3 | 11 (50.0) |  | 34 (54.0) |  | 56 (46.3) | |
| Tested positive for >1 variant | 0 (0.0) | 0.0 | 5 (16.7) | 12.2 | 5 (12.2) | 1 (10.0) | 4.5 | 1 (8.3) | 4.5 | 2 (9.1) |  | 7 (11.1) |  | 9 (7.4) | |
| *CFH* | 3 (27.3) | 7.3 | 10 (33.3) | 24.4 | 13 (31.7) | 2 (20.0) | 9.1 | 3 (25.0) | 13.6 | 5 (22.7) |  | 18 (28.6) |  | 27 (22.3) | |
| *CD46* | 0 (0.0) | 0.0 | 7 (23.3) | 17.1 | 7 (17.1) | 1 (10.0) | 4.5 | 3 (25.0) | 13.6 | 4 (18.2) |  | 11 (17.5) |  | 19 (15.7) | |
| *C3* | 1 (9.1) | 2.4 | 3 (10.0) | 7.3 | 4 (9.8) | 1 (10.0) | 4.5 | 1 (8.3) | 4.5 | 2 (9.1) |  | 6 (9.5) |  | 11 (9.1) | |
| *CFI* | 0 (0.0) | 0.0 | 4 (13.3) | 9.8 | 4 (9.8) | 0 (0.0) | 0.0 | 0 (0.0) | 0.0 | 0 (0.0) |  | 4 (6.3) |  | 6 (5.0) | |
| *DGKE* | 0 (0.0) | 0.0 | 0 (0.0) | 0.0 | 0 (0.0) | 1 (10.0) | 4.5 | 0 (0.0) | 0.0 | 1 (4.5) |  | 1 (1.6) |  | 3 (2.5) | |
| *THBD* | 0 (0.0) | 0.0 | 0 (0.0) | 0.0 | 0 (0.0) | 0 (0.0) | 0.0 | 0 (0.0) | 0.0 | 0 (0.0) |  | 0 (0.0) |  | 1 (0.8) | |
| *CFB* | 0 (0.0) | 0.0 | 0 (0.0) | 0.0 | 0 (0.0) | 0 (0.0) | 0.0 | 0 (0.0) | 0.0 | 0 (0.0) |  | 0 (0.0) |  | 1 (0.8) | |
| Anti-CFH Ab | 1 (9.1) | 2.4 | 7 (23.3) | 17.1 | 8 (19.5) | 2 (20.0) | 9.1 | 0 (0.0) | 0.0 | 2 (9.1) |  | 10 (15.9) |  | 14 (11.6) | |

CFH, complement factor H; n/%, number/percentage of patients in a given category; N, number of patients; Ab, antibody; CD46, membrane cofactor protein; C3, complement component 3; CFI, complement factor I; DGKE, diacylglycerol kinase epsilon; THBD, thrombomodulin; CFB, complement factor B

**Online Resource 4**

Complement gene variants and anti-CFH antibody seropositivity at initial presentation by age (never-treated group)

|  | **Never-treated with eculizumab** | | | | | | | | | | | | | **Treated and never-treated** |
| --- | --- | --- | --- | --- | --- | --- | --- | --- | --- | --- | --- | --- | --- | --- |
|  | **Initial presentation in adulthood** | | | | | **Initial presentation in childhood** | | | | |  | **Adults and children** |  | **Adults and children** |
| **Variable, n (%)** | **Male** | | **Female** | | **Total** | **Male** | | **Female** | | **Total** |  |  |  |  |
|  | **N=18** | **% of all adults** | **N=18** | **% of all adults** | **N=36** | **N=12** | **% of all children** | **N=10** | **% of all children** | **N=22** |  | **N=58** |  | **N=121** |
| Tested for ≥5 variants | 12 (66.7) | 33.3 | 9 (50.0) | 25.0 | 21 (58.3) | 6 (50.0) | 27.3 | 6 (60.0) | 27.3 | 12 (54.5) |  | 33 (56.9) |  | 80 (66.1) |
| Tested for any variant (≥1) or anti-CFH Ab | 16 (88.9) | 44.4 | 15 (83.3) | 41.7 | 31 (86.1) | 10 (83.3) | 45.5 | 8 (80.0) | 36.4 | 18 (81.8) |  | 49 (84.5) |  | 108 (89.3) |
| Tested positive for ≥1 variant | 4 (22.2) | 11.1 | 7 (38.9) | 19.4 | 11 (30.6) | 6 (50.0) | 27.3 | 5 (50.0) | 22.7 | 11 (50.0) |  | 22 (37.9) |  | 54 (44.6) |
| Tested positive for ≥1 variant or anti-CFH Ab | 4 (22.2) | 11.1 | 7 (38.9) | 19.4 | 11 (30.6) | 6 (50.0) | 27.3 | 5 (50.0) | 22.7 | 11 (50.0) |  | 22 (37.9) |  | 56 (46.3) |
| Tested positive for >1 variant | 1 (5.6) | 2.8 | 1 (5.6) | 2.8 | 2 (5.6) | 0 (0.0) | 0.0 | 0 (0.0) | 0.0 | 0 (0.0) |  | 2 (3.4) |  | 9 (7.4) |
| *CFH* | 2 (11.1) | 5.6 | 3 (16.7) | 8.3 | 5 (13.9) | 1 (8.3) | 4.5 | 3 (30.0) | 13.6 | 4 (18.2) |  | 9 (15.5) |  | 27 (22.3) |
| *CD46* | 1 (5.6) | 2.8 | 2 (11.1) | 5.6 | 3 (8.3) | 3 (25.0) | 13.6 | 2 (20.0) | 9.1 | 5 (22.7) |  | 8 (13.8) |  | 19 (15.7) |
| *C3* | 1 (5.6) | 2.8 | 2 (11.1) | 5.6 | 3 (8.3) | 1 (8.3) | 4.5 | 1 (10.0) | 4.5 | 2 (9.1) |  | 5 (8.6) |  | 11 (9.1) |
| *CFI* | 0 (0.0) | 0.0 | 1 (5.6) | 2.8 | 1 (2.8) | 1 (8.3) | 4.5 | 0 (0.0) | 0.0 | 1 (4.5) |  | 2 (3.4) |  | 6 (5.0) |
| *DGKE* | 0 (0.0) | 0.0 | 0 (0.0) | 0.0 | 0 (0.0) | 1 (8.3) | 4.5 | 1 (10.0) | 4.5 | 2 (9.1) |  | 2 (3.4) |  | 3 (2.5) |
| *THBD* | 0 (0.0) | 0.0 | 0 (0.0) | 0.0 | 0 (0.0) | 1 (8.3) | 4.5 | 0 (0.0) | 0.0 | 1 (4.5) |  | 1 (1.7) |  | 1 (0.8) |
| *CFB* | 1 (5.6) | 2.8 | 0 (0.0) | 0.0 | 1 (2.8) | 0 (0.0) | 0.0 | 0 (0.0) | 0.0 | 0 (0.0) |  | 1 (1.7) |  | 1 (0.8) |
| anti-CFH Ab | 1 (5.6) | 2.8 | 1 (5.6) | 2.8 | 2 (5.6) | 0 (0.0) | 0.0 | 2 (20.0) | 9.1 | 2 (9.1) |  | 4 (6.9) |  | 14 (11.6) |

CFH, complement factor H; n/%, number/percentage of patients in a given category; N, number of patients; Ab, antibody; CD46, membrane cofactor protein; C3, complement component 3; CFI, complement factor I; DGKE, diacylglycerol kinase epsilon; THBD, thrombomodulin; CFB, complement factor B

**Online Resource 5**

Disease characteristics according to selected potential precipitating factors prior to aHUS diagnosis

|  |  | **Group** |  | **Prior transplant** | **Malignancy** | **Pregnancy** | **Autoimmune disease** | **Malignant hypertension** |
| --- | --- | --- | --- | --- | --- | --- | --- | --- |
| **Eculizumab-treated** |  | Male   N=11 | n (%) | 0 | 0 | 0 | 0 | 1 (9.1) |
|  | Initial presentation in adulthood |  | % of total adults | 0 | 0 | 0 | 0 | 2.4 |
|  |  | Female   N=30 | n (%) | 3 (10.0) | 0 | 5 (16.7) | 1 (3.3) | 3 (10.0) |
|  |  |  | % of total adults | 7.3 | 0 | 12.2 | 2.4 | 7.3 |
|  |  | Total   N=41 | n (%) | 3 (7.3) | 0 | 5 (12.2) | 1 (2.4) | 4 (9.8) |
|  | Initial presentation in childhood | Male   N=10 | n (%) | 0 | 0 | 0 | 0 | 0 |
|  |  |  | % of total children | 0 | 0 | 0 | 0 | 0 |
|  |  | Female   N=12 | n (%) | 0 | 0 | 0 | 0 | 0 |
|  |  |  | % of total children | 0 | 0 | 0 | 0 | 0 |
|  |  | Total   N=22 | n (%) | 0 | 0 | 0 | 0 | 0 |
|  | **Total (adults and children) N=63** | | n (%) | 3 (4.8) | 0 | 5 (7.9) | 1 (1.6) | 4 (6.3) |
| **Non-treated** | Initial presentation in adulthood | Male   N=18 | n (%) | 3 (16.7) | 1 (5.6) | 0 | 0 | 0 |
|  |  |  | % of total adults | 8.3 | 2.8 | 0 | 0 | 0 |
|  |  | Female   N=18 | n (%) | 2 (11.1) | 0 | 1 (5.6) | 1 (5.6) | 0 |
|  |  |  | % of total adults | 5.6 | 0 | 2.8 | 2.8 | 0 |
|  |  | Total   N=36 | n (%) | 5 (13.9) | 1 (2.8) | 1 (2.8) | 1 (2.8) | 0 |
|  | Initial presentation in childhood | Male   N=12 | n (%) | 0 | 0 | 0 | 0 | 0 |
|  |  |  | % of total children | 0 | 0 | 0 | 0 | 0 |
|  |  | Female   N=10 | n (%) | 0 | 0 | 0 | 0 | 3 (30.0) |
|  |  |  | % of total children | 0 | 0 | 0 | 0 | 13.6 |
|  |  | Total   N=22 | n (%) | 0 | 0 | 0 | 0 | 3 (13.6) |
|  |  | **Total (adults and children) N=58** | n (%) | 5 (8.6) | 1 (1.7) | 1 (1.7) | 1 (1.7) | 3 (5.2) |
| **Treated and non-treated (adults and children)** |  | **Total N=121** | n (%) | 8 (6.6) | 1 (0.8) | 6 (5.0) | 2 (1.7) | 7 (5.8) |

aHUS, atypical hemolytic uremic syndrome; N, number of patients; n/%, number/percentage of patients in a given group

**Online Resource 6**

Multivariable Cox regression analysis for risk factor association with KF (N=111^a^)

|  |  | **n** | **KF events, n** | **Unadjusted HR**  **(95% CI)** | **Adjusted HR**  **(95% CI)** |
| --- | --- | --- | --- | --- | --- |
| Age at onset | Adult | 69 | 29 | 1.0 | 1.0 |
|  | Pediatric | 42 | 11 | 0.3 (0.2–0.7) | 0.2 (0.1–0.6) |
| Gender | Female | 63 | 22 | 1.0 | 1.0 |
|  | Male | 48 | 18 | 0.8 (0.4–1.5) | 1.3 (0.6–3.0) |
| Race | White | 101 | 36 | 1.0 | 1.0 |
|  | Non-White | 10 | 4 | 1.1 (0.4–3.0) | 0.7 (0.1–5.6) |
| Family history of aHUS | No | 73 | 22 | 1.0 | 1.0 |
|  | Yes | 15 | 9 | 1.0 (0.4–2.5) | 1.1 (0.5–2.8) |
| Time from onset to diagnosis, days | 0 | 36 | 12 | 1.0 | 1.0 |
|  | 1–14 | 53 | 16 | 1.5 (0.7–3.4) | 1.7 (0.6–4.7) |
|  | 15–30 | 7 | 4 | 4.3 (1.3–14.6) | 4.1 (1.0–16.8) |
|  | 31–180 | 7 | 4 | 2.5 (0.8–8.2) | 2.7 (0.6–11.9) |
|  | >180 | 8 | 4 | 0.9 (0.3–3.0) | 0.5 (0.1–2.4) |
| Any precipitating factor | No | 98 | 33 | 1.0 | 1.0 |
|  | Yes | 13 | 7 | 1.6 (0.7–3.6) | 1.1 (0.4–3.4) |

KF, kidney failure; N, number of patients; n, number of patients in a given group; HR, hazard ratio; CI, confidence interval; aHUS, atypical hemolytic uremic syndrome

^a^Ten patients were excluded from this analysis due to negative values of time-to-KF

Adjusted HRs are derived from full regression model including all covariates shown in the table. Adult group is the reference group - HR >1 suggests an increased risk; HR <1 suggests a lower risk compared to the reference group.
